# A Dietary Assessment App for Hospitalized Patients at Nutritional Risk: Development and Evaluation of the MyFood App

**DOI:** 10.2196/mhealth.9953

**Published:** 2018-09-07

**Authors:** Mari Mohn Paulsen, Martina Lovise Lindhart Hagen, Marte Hesvik Frøyen, Rikke Julie Foss-Pedersen, Dagfinn Bergsager, Randi Julie Tangvik, Lene Frost Andersen

**Affiliations:** ^1^ National Advisory Unit on Disease-Related Malnutrition Division of Cancer Medicine Oslo University Hospital Oslo Norway; ^2^ Institute of Basic Medical Sciences Department of Nutrition University of Oslo Oslo Norway; ^3^ The University Center for Information Technology University of Oslo Oslo Norway; ^4^ Department of Clinical Medicine Faculty of Medicine University of Bergen Bergen Norway

**Keywords:** decision support system, disease-related malnutrition, eHealth, mHealth, dietary assessment, validation study

## Abstract

**Background:**

Disease-related malnutrition is a common challenge among hospitalized patients. There seems to be a lack of an effective system to follow-up nutritional monitoring and treatment of patients at nutritional risk after risk assessment. We identify a need for a more standardized system to prevent and treat disease-related malnutrition.

**Objective:**

We aimed to develop a dietary assessment app for tablets for use in a hospital setting and to evaluate the app’s ability to measure individual intake of energy, protein, liquid, and food and beverage items among hospitalized patients for two days. We also aimed to measure patients’ experiences using the app.

**Methods:**

We have developed the MyFood app, which consists of three modules: 1) collection of information about the patient, 2) dietary assessment function, and 3) evaluation of recorded intake compared to individual needs. We used observations from digital photography of the meals, combined with partial weighing of the meal components, as a reference method to evaluate the app’s dietary assessment system for two days. Differences in the intake estimations of energy, protein, liquid, and food and beverage items between MyFood and the photograph method were analyzed on both group and individual level.

**Results:**

Thirty-two patients hospitalized at Oslo University Hospital were included in the study. The data collection period ran from March to May 2017. About half of the patients had ≥90% agreement between MyFood and the photograph method for energy, protein, and liquid intake on both recording days. Dinner was the meal with the lowest percent agreement between methods. MyFood overestimated patients’ intake of bread and cereals and underestimated fruit consumption. Agreement between methods increased from day 1 to day 2 for bread and cereals, spreads, egg, yogurt, soup, hot dishes, and desserts. Ninety percent of participants reported that MyFood was easy to use, and 97% found the app easy to navigate.

**Conclusions:**

We developed the MyFood app as a tool to monitor dietary intake among hospitalized patients at nutritional risk. The recorded intake of energy, protein, and liquid using MyFood showed good agreement with the photograph method for the majority of participants. The app’s ability to estimate intake within food groups was good, except for bread and cereals which were overestimated and fruits which were underestimated. The app was well accepted among study participants and has the potential to be a dietary assessment tool for use among patients in clinical practice.

## Introduction

Disease-related malnutrition is a common challenge in patients with chronic or severe diseases [1] with a prevalence of 30%-50% in hospitals [1-7]. Malnutrition has several health-related consequences for patients. It increases morbidity and mortality [1,2,8-10], length of stay [2,3,9,11,12], and readmission rates [2]. Disease-related malnutrition has significant economic consequences for the health care system [8,12-13].

According to the Norwegian “National guidelines for prevention and treatment of malnutrition” [14] and European guidelines recommended by the European Society of Clinical Nutrition and Metabolism [15], all patients should be screened for nutritional risk upon admission to hospital and weekly thereafter. Information on the patient’s nutritional status and treatment should be documented in medical records and communicated to the next level of care. All patients at nutritional risk should have an individual nutrition plan including documentation of nutritional status, needs, dietary intake, and recommended actions. Hospitals, nursing homes, and home care services are responsible for integrating nutrition in the care and treatment of all patients [14].

European data from the nutritionDay survey indicates that dietary assessment is only performed for a small number of patients at nutritional risk and that documentation of food intake is rarely done [16]. Norwegian studies have reported that about half [17] or fewer than half [7] of patients identified to be at nutritional risk receive nutritional treatment. A barrier to adequate nutritional care for malnourished patients in hospitals is the absence of routines, as demonstrated in qualitative studies among nurses in Norway and Sweden [18,19]. Nurses report a lack of tools to estimate patients’ needs and the content of energy and protein in hospital menus [11]. They also report insufficient knowledge and skills to identify and treat malnourished patients [18,20].

In the next decade, the need for healthcare will increase and, there will be a shortage of labor. This should be met with more effective, less people-demanding services and increased use of welfare technology [21]. There seems to be a lack of an effective system to follow up nutritional treatment in the healthcare system. We have identified a need for a more standardized system for prevention and treatment of disease-related malnutrition. To the best of our knowledge, no studies regarding development of an electronic decision support system for prevention and treatment of disease-related malnutrition among hospitalized patients at nutritional risk have been performed.

We developed an app, MyFood (MinMat), for mini tablet computers as part of a decision support system to prevent and treat disease-related malnutrition. Assessment in the app is based on self-reported dietary intake where the patient (or a nurse) records consumption of food and beverages. The memory of intake, ability to estimate portion sizes, and perceptions of socially desirable responses are well-known challenges associated with self-reported dietary intake [22]. Self-reported methods for assessment of dietary intake have been found to underestimate energy intake by approximately 20% when compared to doubly labeled water [23-25]; dietary assessment methods should always be validated because of these methodological challenges [22]. Therefore, evaluation of MyFood’s ability to track the patients’ dietary intake is of crucial importance.

The aim of this study was to develop a dietary assessment app for tablets for use in a hospital setting and to evaluate the app’s ability to measure individual intake of energy, protein, liquid, and food and beverages for two days compared to photograph observations combined with partial weighing as the reference method. We also aimed to measure the patients’ experiences using the app.

## Methods

### Development of the MyFood App

My Food was developed by researchers at the University of Oslo and Oslo University Hospital (OUH) and by interaction designers and developers at the University Center for Information Technology (USIT).

Nurses and patients were involved in the design process. Paper sketches of MyFood were developed and explored with three nurses and three patients at the Department of Gastrointestinal Surgery at OUH, Rikshospitalet. The feedback we received was used to modify the design and content of the app before the technical development process began. A prototype of MyFood was then developed and tested by four patients and two nurses. Their feedback was used for additional modifications of MyFood before the evaluation study was performed.

MyFood consisted of the following three modules:

#### Module 1: Collection of Information About the Patient

In the first module, the nurse, or other healthcare professional, recorded information about the patient. This information included: Norwegian patient registry (NPR) number, gender, date of birth, height (in centimeters), weight (in kilograms), whether the patient had a fever (and, if so, the number of degrees, and whether the patient was following a special diet or had any special preferences with regard to food or beverages.

#### Module 2: Dietary Assessment Function

**Figure 1:**
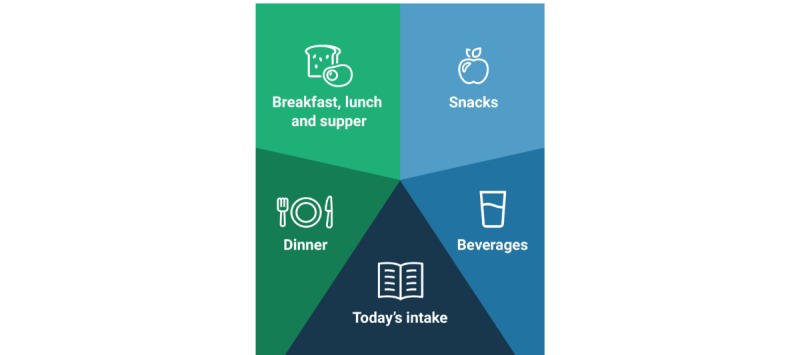
The main menu of dietary recording in the MyFood app.

Figure 1 shows the main menu in the dietary assessment function in MyFood.

**Figure 2:**
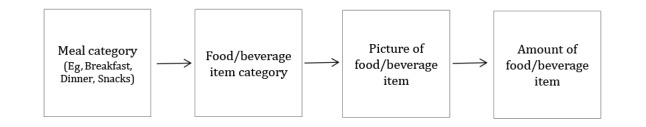
Flowchart on the dietary recording function in module 2.

Recording of food intake was done by first selecting the relevant meal category and then selecting the category for the food or beverage item. The food and beverage categories included pictures of the different items. Pictures could also be found using free text search. After selecting the food or beverage item consumed, the item amount was recorded. Portion size could be selected with a precision of a half unit. Figure 2 is a flowchart of dietary recording in the app. Multimedia Appendix 1 shows some selected print screens from MyFood.

**Figure 3:**
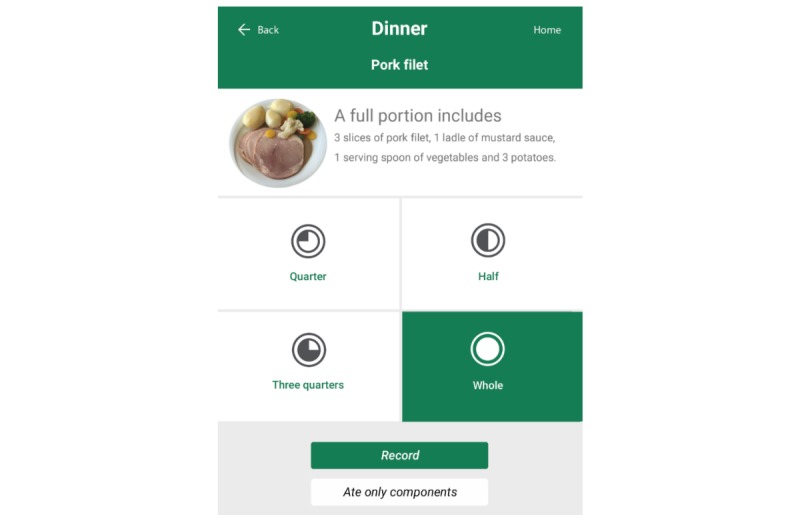
Recording of hot dishes in MyFood.

Intake of energy, protein, and liquid was calculated based on information of the nutrient content in standard units (eg, 1 slice of bread, 1/2 glass of milk). The app included prompting questions (eg, regarding the use of spreads when recording intake of bread or regarding intake of beverages together with meals). Hot dishes were recorded by selecting an icon depicting the portion consumed (full, three quarters, half, one quarter; Figure 2). If only components of the meal were consumed (eg, 1 potato) this could be recorded by choosing the “ate only components” function shown in Figure 3. Portion sizes for beverages were recorded by selecting an icon depicting sections of a glass/cup (full, three quarters, half, one quarter) or by inputting the number of deciliters consumed.

The app included pictures of all food and beverages served at OUH, Rikshospitalet. It also included pictures of different groceries, food, and beverages that may be brought by relatives or friends from outside the hospital as well as advanced medical nutrition products. Nutritional information in the app was given for the intake of energy (kcal), protein (grams), and liquid (milliliters). Nutritional data were retrieved from an in-house data program (KBS version 7.0), based on the Norwegian food composition table [26], and from manufacturers.

#### Module 3: Evaluation of Recorded Intake Compared to Individual Needs

The third module automatically compared dietary intake with individual requirements for energy, protein, and liquids. This module was developed by including several algorithms in the app. The algorithms estimated the patients’ daily requirements for energy, protein, and liquids and were based on recommendations from the Norwegian Directorate of Health [14,27].

### Technological Features

**Figure 4:**
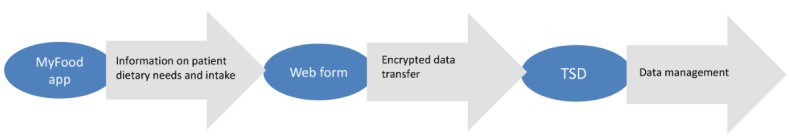
Data flow in the MyFood app. TSD: services for sensitive data.

The data flow in the app used a Web form and secure storage in “Services for sensitive data” (or TSD, Tjenester for Sensitive Data) [28] hosted by USIT (Figure 4). TSD meets the stringent requirements for the processing and storage of sensitive research data and is included in NorStore, the Norwegian national infrastructure for handling and storage of scientific data [29]. All recorded data were sent to TSD continuously during the data collection period. The recorded data were also stored locally on the iPad and visible in the app until 3 am the following day. This made it possible for the respondents to edit their recordings of dietary intake and the app was able to give the users feedback on their intake of energy, protein, and liquid during the current day. If the iPad was not able to send the data to TSD (eg, missing internet connection), the data were encrypted, temporarily queued, and resent as soon as the iPad was online again. All iPads were “clean” every morning and could possibly be given to a new patient. The data were later retrieved from TSD for data analysis in the evaluation study.

The Mobile Device Management System, AirWatch, was used to control the iPads during the data collection period. If tablets disappeared, we were able to clean the disappeared tablet remotely and make it impossible to use until reopened via AirWatch. It was possible to maintain total control of sensitive data stored on the tablets using this system.

### Evaluation of the MyFood App

#### Participants

The evaluation study was performed at OUH, Rikshospitalet in the Departments of Gastrointestinal Surgery and Hematology.

Inclusion criteria were:

- ≥18 years of age
- ≥2 days of expected stay

Exclusion criteria were:

- Pregnancy
- Special infection precautions
- Psychiatric patients
- Critically ill patients
- Patients not able to read the Norwegian language

Even though MyFood is designed to be used by patients at nutritional risk, this was not an inclusion criterion as we wanted to include patients eating various amounts of food to evaluate the app. Based on a power of 0.8, a significance level of *P*=.05 and a calculated standardized difference of 1.0, 32 patients were included in the evaluation study.

#### Ethics

The study was performed in accordance with the Helsinki declaration and was acknowledged by the Norwegian Regional ethical committee (2016/1464), the Data protection officer at OUH, and the Chief Information Security Officer at the University of Oslo. Informed written consent was collected from all participating patients.

#### Performance

Information about the study, including the nurses’ responsibilities in the data collection period, was sent to all nurses via e-mail. In the Department of Hematology, a ten-minute presentation by the project workers was held for the nurses during the morning meetings of the first two days of the data collection period. The responsible nurse in each department identified patients who met all of the inclusion criteria and none of the exclusion criteria.

The patients were registered in the app by a nurse or by one of the project workers before breakfast. Information including the NPR number, height, weight, presence of fever, special diet, or special preferences was registered in the app before patient use.

Written instructions on how to record dietary intake in MyFood were given to the patients and the nurses. Once included in the study, patients answered a form with information about education, living conditions, and level of experience with apps and tablets or smartphones.

Included patients were given a tablet (iPad mini 32GB) and were asked to use MyFood for two days to record their intake of food and beverages for the breakfast, lunch, and dinner meals. If patients were not able to or did not want to record information themselves, a nurse performed the dietary recording for them. The patients were instructed to record dietary intake as soon as possible after the meals in order to get as precise recordings as possible. They were also informed both verbally and in writing to record the intake for the breakfast, lunch, and dinner meals only, not snacks or beverages consumed between the respective meals. If patients did not find exactly what they had consumed in the app, they were instructed to record something similar.

After two days of using MyFood, participants were asked to answer a form regarding assumptions about comprehension, content, and perceived value and usability of the app.

#### Reference Method

We used observations from digital photography of the meals combined with partial weighing of meal components as the reference method to evaluate the dietary assessment function in MyFood. The reference method is further described as the photograph method. A digital system camera (Sony A500/16-50mm PZ objective) was mounted to a removable trolley (85 cm * 50 cm) on an adjustable and pivotable tripod. The camera lens was approximately 0.6 m above the trolley. A researcher photographed the trays with the patients’ meal before and after consumption. The trays were marked with the study participant number. The numbered trays were placed on a marked area on the trolley and a 30-cm ruler was placed on the tray as a reference size. The photographs were taken at an angle of 45° to the trays so that in-depth images could be taken for more convenient meal content estimation. In addition to the observations from photographs, partial weighing of meal components was performed by the researchers. Plates, glasses, cups, and food items in separate packaging were weighed on an electronic scale before and after the meal. In cases where determining the type of food or beverage from the photographs were challenging (eg, whole fat or skimmed milk, sugar-sweetened or light soft drinks, butter or margarine), the patients were asked about what specific type of foods or beverages they included in the meal.

#### Training of Project Workers

Two project workers underwent practice in photographing and estimating portion sizes before the data collection, to secure a standardized method and higher level of agreement. Thirteen meals (both bread-based meals and hot dishes) were prepared by a third person. The meals were prepared to illustrate the portion size before consumption and after consumption, by removing all or parts of the food. The meals were photographed before and after some or all the food were removed from the tray. Glasses, plates, cups, and food items in separate packaging were weighed. Both project workers observed the photographs and calculated the weights to estimate the consumption of food and beverages. The interobserver reliability (IOR) between the two project workers was calculated to be 0.92 for energy content. The project workers’ estimations of energy content matched with the known energy content by 0.94. This was considered satisfactory, based on criteria in other studies [30].

#### Data Handling and Statistical Analyses

The food and beverage intake observed from the photographs and estimated from partly weighing in the evaluation study were compared with the intake recorded in MyFood. Observed and weighed intake was estimated separately by the two project workers, before recording the data in an in-house diet calculation system (KBS version 7.0). The project workers estimations were compared with the requirement of an IOR above 0.85 for energy, protein, and liquids in each meal. If IOR was <0.85 the calculations were repeated and recompared. In cases with obvious typing mistakes, this was corrected by the respective project worker. If the project workers had estimated different amounts, the pictures were re-evaluated, and the project workers agreed on where to adjust the estimated amounts (in grams). After corrections, the total IOR was 0.97 for energy, 0.98 for protein, and 0.98 for liquid. A final data file with estimated consumption based on the photograph method was created by averaging the estimations of the two project workers.

Statistical analyses were performed using the statistical software package IBM SPSS Statistics 24. All tests were two-sided with a 5% level of significance. The data were analyzed on both group and individual level. Differences in the intake estimations of energy, protein, liquid, and food groups, between MyFood and the photograph method, were analyzed with Wilcoxon Signed Ranks Test due to nonnormally distributed variables. Multiple scatter plots of consumption of energy, protein, liquid, and selected food groups were used to illustrate the difference between the estimated intake in MyFood and from the photograph method for each individual subject. The differences between the methods were assessed in total and divided into the breakfast, lunch, and dinner meals for recording days 1 and 2 separately. To calculate omitted food items, one omission was counted as an item observed from photographs in a meal but not recorded in MyFood.

## Results

### Participants

**Figure 5:**
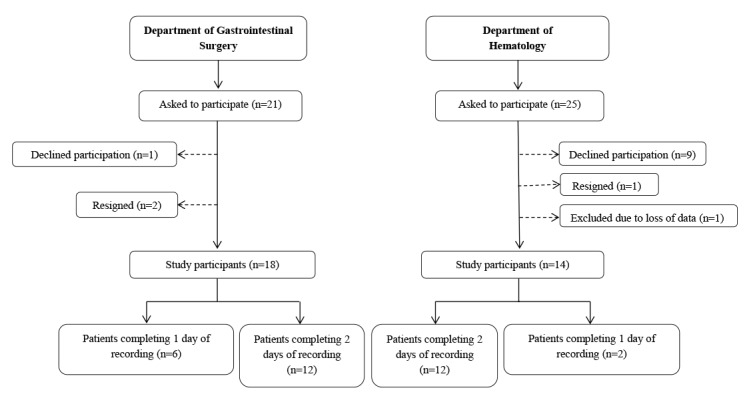
Flowchart of the recruitment process of study participants.

The study sample consisted of 32 patients at OUH, Rikshospitalet; 18 from the Department of Gastrointestinal Surgery and 14 from the Department of Hematology. The data collection period ran from March to May 2017, and the participants were recruited continuously during the period. A flowchart describing the recruitment process is illustrated in Figure 5.

Characteristics of the study participants are illustrated in Table 1. More than two-thirds were men and the age distribution was from 17 to 77 years. About 40 percent of the participants were characterized as normal weight, according to body mass index (BMI) and more than half as overweight or obese. The majority of the participants had some or a lot of experience with apps and smartphones or tablets.

### Estimations of Energy, Protein, and Liquid Consumption in MyFood Compared to the Photograph Method on Group Level

Table 2 shows the intake of energy, protein, and liquids estimated in MyFood and the photograph method. The results are presented for the total of breakfast, lunch, and dinner, and separately for each meal.

The median intake of energy was not significantly different between the methods the first day, except for lunch where median recorded intake in MyFood was significantly higher compared to the photograph method. The second day a significantly lower median total energy intake was found in MyFood compared to the photograph method. The opposite was observed for the lunch and dinner meal.

The recorded median protein intake in MyFood was significantly lower for total intake, breakfast, and lunch, compared to the photograph method on day 1. The second day the median intake of protein for breakfast was significantly lower in MyFood, compared to the photograph method. No other statistically significant differences were found for median protein intake on day 2.

The median liquid intake showed relatively good agreement between the methods on the group level. Only for breakfast the first day the median recorded intake was significantly lower in MyFood compared to the photograph method.

### Estimations of Energy, Protein, and Liquid Consumption in MyFood Compared to the Photograph Method on Individual Level

Table 3 shows the percentage of the patients who had 90 and 80 percent agreement between their recordings in MyFood compared to the photograph method, in total and separately for the breakfast, lunch, and dinner meal.

About half of the patients had ≥90% agreement in total for energy, protein, and liquid intake, somewhat lower for protein and higher for liquids both recording days. The breakfast meal had the highest proportion of participants with ≥80% agreement between the methods for all nutrient components both days, except for protein intake the first recording day. The agreement between the methods was lowest for the dinner meal.

#### Energy intake

**Figure 6:**
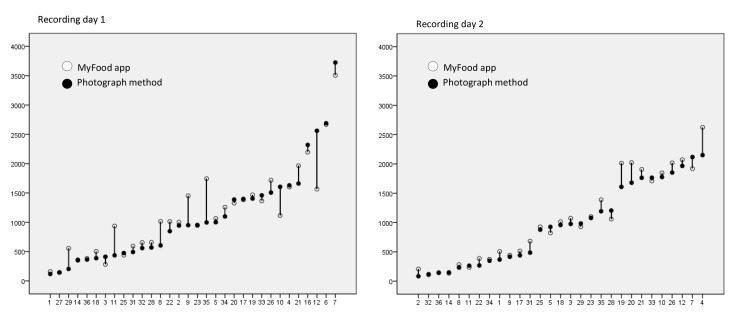
Drop plots illustrating individual intake of energy recording day 1 (n=32) and recording day 2 (n=29). Y-axis represents energy intake (kcal). X-axis represents participant number ranged with increasing energy intake according to the photograph method. Equal energy intake from app and photograph observations is presented with only black dots.

Recorded individual energy intake in MyFood and intake estimated from the photograph method are illustrated in Figure 6, which shows individual drop-plots from the first and second recording days.

MyFood estimated the energy consumption relatively accurate for the majority of the patients. On average for the two days, approximately 70% of the participants had less than 20% disagreement between the two methods, and approximately 50% had less than 10% disagreement (Table 3). For some participants, the intake was overestimated in MyFood compared to photograph observations (Figure 6). This overestimation was more pronounced on day 1 than day 2. The largest discrepancies with regard to energy consumption at the individual level were found for the dinner meal the first day (Table 3).

**Table 1:** Characteristics of participants (n=32) in the evaluation study of MyFood.

Characteristic		n (%)
**Hospital department**		
	Gastrointestinal surgery	18 (56)
	Hematology	14 (44)
**Gender**		
	Men	22 (69)
	Women	10 (31)
**Age (years)^a^**		
	<30	3 (10)
	30-39	3 (10)
	40-49	7 (23)
	50-59	8 (26)
	60-69	8 (26)
	70-80	2 (7)
**Body mass index (kg/m^2^)**		
	<18.5	1 (3)
	18.5-24.9	13 (41)
	25-29.9	14 (44)
	>30	4 (13)
**Education**		
	Primary and secondary schools	4 (13)
	Comprehensive school/high school	16 (50)
	College/university ≤4 years	6 (19)
	College/university >4 years	6 (19)	
**Earlier experiences with apps and smartphones/tablets**		
	None/little	3 (9)
	Some (use sometimes)	9 (28)
	A lot (use often/daily)	20 (63)

^a^Missing n=1.

#### Protein Intake

The individual protein consumption recorded in MyFood, compared to the photograph method showed relatively coinciding agreement. The agreement was most coinciding on day 2 (Multimedia Appendix 2). On average for the two days, about 70% of the participants had less than 20% disagreement between the two methods, and just below half of the participants had less than 10% disagreement (Table 3). The discrepancy between the methods was largest for the dinner meal (Table 3).

#### Liquid Intake

The agreement between the methods for low and medium liquid intake was good, with a tendency to increased deviations for higher intakes. This was seen on both recording days (Multimedia Appendix 3). On average for the two days, about 60%-70% of the participants had less than 20% disagreement in liquid intake between the two methods, and about 50% had less than 10% disagreement (Table 3).

### Estimations of Food Intake in MyFood Compared to the Photograph Method on Group Level

The consumption (grams) within food groups are shown in Table 4. No statistically significant differences were seen between the methods, except for bread and cereals, and fruits. The median recorded intake of bread and cereals was significantly higher in MyFood compared to the photograph method, both recording days. Median fruit intake was significantly lower in MyFood the first recording day.

**Table 2:** Energy, protein, and liquid consumption recorded in MyFood compared to the photograph method. The data are presented as a total of the breakfast, lunch, and dinner meals, and separately for each meal.

Energy, protein and liquid				Mean	Median (25-75 percentile)	*P* value
**Energy**						
	**Day 1**					
		**Breakfast (n=28)**				.73^a^
			MyFood	471	398 (244-616)	
			Photograph method	458	373 (222-373)	
		**Lunch (n=27)**				.04^a^
			MyFood	408	389 (262-494)	
			Photograph method	382	308 (201-308)	
		**Dinner (n=27)**				.58^a^
			MyFood	476	468 (210-711)	
			Photograph method	461	477 (226-575)	
		**Total (n=32)**				.11^a^
			MyFood	1157	1039 (556-1541)	
			Photograph method	1102	951 (446-1495)	
	**Day 2**					
		**Breakfast (n=29)**				.74^a^
			MyFood	400	374 (223-527)	
			Photograph method	407	367 (175-630)	
		**Lunch (n=20)**				.02^a^
			MyFood	454	501 (258-608)	
			Photograph method	394	418 (245-514)	
		**Dinner (n=20)**				.01^a^
			MyFood	489	413 (134-820)	
			Photograph method	425	368 (105-696)	
		**Total (n=29)**				.009^a^
			MyFood	1050	928 (380-1876)	
			Photograph method	972	957 (308-1720)	
**Protein (g)**						
	**Day 1**					
		**Breakfast (n=28)**				.02
			MyFood	16.2	13.5 (6.4-23.5)	
			Photograph method	18.2	14.3 (6.9-27.7)	
		**Lunch (n=27)**				.001
			MyFood	13.0	10.0 (8.0-18.0)	
			Photograph method	14.6	13.1 (7.8-20.2)	
		**Dinner (n=27)**				.22
			MyFood	15.2	14.5 (3.0-20.5)	
			Photograph method	16.8	14.3 (6.1-22.8)	
		**Total (n=32)**				.046
			MyFood	38.0	35.0 (17.4-45.6)	
			Photograph method	42.2	38.4 (14.0-62.1)	
	**Day 2**					
		**Breakfast (n=29)**				<.001
			MyFood	14.1	11.0 (5.0-19.8)	
			Photograph method	16.1	15.8 (5.7-22.0)	
		**Lunch (n=20)**				.31
			MyFood	14.2	13.3 (5.9-20.3)	
			Photograph method	12.9	11.5 (7.8-16.7)	
		**Dinner (n=20)**				.97
			MyFood	17.6	15.3 (5.3-30.4)	
			Photograph method	17.6	16.5 (3.9-28.7)	
		**Total (n=29)**				.15
			MyFood	36.1	28.0 (8.5-61.5)	
			Photograph method	37.1	34.6 (9.1-61.4)	
**Liquid (ml)**						
	**Day 1**					
		**Breakfast (n=28)**				.02
			MyFood	292	272 (158-412)	
			Photograph method	339	320 (202-466)	
		**Lunch (n=27)**				.72
			MyFood	287	256 (194-409)	
			Photograph method	285	257 (159-374)	
		**Dinner (n=27)**				.33
			MyFood	336	304 (189-304)	
			Photograph method	332	326 (222-445)	
		**Total (n=32)**				.71
			MyFood	781	696 (479-1047)	
			Photograph method	808	643 (461-1227)	
	**Day 2**					
		**Breakfast (n=29)**				.97
			MyFood	301	287 (169-429)	
			Photograph method	303	312 (162-435)	
		**Lunch (n=20)**				.87
			MyFood	275	251 (142-405)	
			Photograph method	260	256 (154-345)	
		**Dinner (n=20)**				.06
			MyFood	311	269 (84-540)	
			Photograph method	273	245 (58-487)	
		**Total (n=29)**				.11
			MyFood	706	587 (313-1077)	
			Photograph method	670	559 (318-1029)	

^a^Differences between MyFood and the photograph method for the breakfast, lunch and dinner meal. The totals of these meals are tested with Wilcoxon Signed Rank test separately for each recording day.

**Table 3:** Proportions with 90% and 80% agreement between MyFood and the photograph method in estimated intake of energy, protein, and liquids.

Energy, protein and liquid			Percent agreement	
			90%	80%
**Day 1**				
	**Energy**			
		Total (n=32)	47	69
		Breakfast (n=28)	46	79
		Lunch (n=27)	48	59
		Dinner (n=27)	30	52
	**Protein**			
		Total (n=32)	44	66
		Breakfast (n=28)	32	64
		Lunch (n=27)	44	74
		Dinner (n=27)	19	41
	**Liquids**			
		Total (n=32)	53	63
		Breakfast (n=28)	39	68
		Lunch (n=27)	48	63
		Dinner (n=27)	26	44
**Day 2**				
	**Energy**			
		Total (n=29)	55	76
		Breakfast (n=29)	45	66
		Lunch (n=20)	40	55
		Dinner (n=20)	25	50
	**Protein**			
		Total (n=29)	48	83
		Breakfast (n=29)	52	69
		Lunch (n=20)	35	55
		Dinner (n=20)	40	50
	**Liquids**			
		Total (n=29)	59	72
		Breakfast (n=29)	52	69
		Lunch (n=20)	45	65
		Dinner (n=20)	45	55

### Estimations of Food Intake in MyFood Compared to the Photograph Method on Individual Level

Table 5 shows the percentage of the participants who had 90 and 80 percent agreement between their recordings in MyFood compared to the photograph method within food groups. Egg was the food group with the best agreement between MyFood and the photograph method with the majority of the estimations ≥90% agreement. The food groups with the lowest agreement were fruit and vegetables. The agreement between the methods increased from day 1 to day 2 for bread and cereals, spreads, egg, yogurt, soup, hot dishes, and desserts.

Estimated bread and cereal consumption was, in most cases, higher in MyFood compared to estimations from the photograph method (Multimedia Appendix 4). On average for the two days, about 60% of the participants had less than 20% disagreement in estimated bread and cereal intake between the two methods, and about 25% had less than 10% disagreement (Table 5).

Recordings of spreads tended to be lower in MyFood compared to the photograph method when the intake increased (Multimedia Appendix 4). About 70% of the participants had less than 20% disagreement between MyFood and the photograph method in estimated intake of spreads on day 2, compared to 50% on day 1 (Table 5).

The food group with the largest deviations between the methods was hot dishes. The discrepancies were highest the first day (Multimedia Appendix 5). About 30%-40% of the participants had ≥80% agreement between the methods (Table 5).

On average for the two days, about 70% of the participants had less than 20% disagreement in estimated intake of cold beverages between the two methods, and about 50% had less than 10% disagreement (Table 5). No particular pattern in discrepancies of cold beverages between the methods was seen (Multimedia Appendix 5).

### Omitted Food Items in MyFood Recordings Compared to the Photograph Method

The number of food and beverage items recorded in MyFood and observed from photographs was calculated (Multimedia Appendices 6 and 7). The first day the number of medical nutrition drinks, cheese, fish-based spreads, and meat-based spreads recorded had 100% matches between the methods (Multimedia Appendix 6). The second day 100% matches were found for the recordings of hot dishes, medical nutrition drinks, vegetables, and meat-based spreads (Multimedia Appendix 7). Butter, margarine, and mayonnaise (27% omissions both days), fruit (27% omissions on day 1), vegetables (28% omissions on day 1), yogurt (27% omissions on day 2) and meal condiments (29% omissions on day 1, 33% omissions on day 2) were the food groups most often omitted among participants (Multimedia Appendices 6 and 7). Five participants had duplicate recordings of some meal components the first day and one participant the second day.

### Patients’ Experiences Using the MyFood App

Ninety percent of the participants reported that MyFood was easy to use. All but one (97%) of the participating patients found the app easy to navigate in. Most of the patients (87%) experienced to record correct amount of foods and beverages. Thirteen percent had to acquire new knowledge to use the app. Seventy-one percent reported to be become more aware of the amount of foods and beverages needed, after using MyFood.

**Table 4:** Food and beverage intake (grams) recorded in MyFood and estimated in the photograph method. Significant *P* values (<.05) are in italics.

Food and beverages		MyFood (grams)		Photograph methods (grams)		*P* value^a^
		Median (25-75 percentile)	Mean (SD)	Median (25-75 percentile)	Mean (SD)	
**Day 1**						
	Bread and cereals (n=23)	110 (50-180)	134 (106)	93 (44-150)	110 (80)	*<.001*
	Spreads^b^ (n=22)	72 (22-120)	77 (59)	89 (20-135)	90 (74)	.17
	Egg (n=11)	56 (56-72)	61 (34)	56 (56-96)	68 (29)	.18
	Yogurt (n=9)	150 (100-223)	154 (81)	190 (101-292)	186 (94)	.09
	Cold beverages (n=30)	350 (200-463)	360 (216)	400 (203-534)	383 (185)	.35
	Hot beverages (n=9)	200 (149-350)	244 (147)	200 (145-394)	261 (175)	.24
	Oral nutritional supplements (n=3)	200 (0-400)	200 (200)	200 (150-400)	250 (132)	.32
	Soup (n=9)	225 (225-270)	235 (114)	202 (147-307)	219 (119)	.37
	Hot dishes (n=20)	217 (0-419)	222 (212)	238 (80-344)	245 (165)	.58
	Desserts (n=12)	58 (10-80)	49 (32)	57 (27-78)	63 (50)	.86
	Fruit (n=10)	30 (0-94)	44 (49)	91 (57-148)	101 (47)	*.04*
	Vegetables^c^ (n=10)	35 (5-60)	51 (64)	60 (38-103)	76 (50)	.18
**Day 2**						
	Bread and cereals (n=24)	83 (40-159)	100 (67)	72 (30-137)	86 (62)	*<.001*
	Spreads^b^ (n=17)	44 (23-60)	56 (47)	54 (24-81)	64 (50)	.13
	Egg (n=8)	56 (50-56)	67 (60)	56 (50-56)	68 (59)	.32
	Yogurt (n=8)	150 (75-150)	118 (63)	145 (90-170)	131 (50)	.78
	Cold beverages (n=25)	300 (200-600)	362 (247)	335 (183-559)	358 (239)	.88
	Hot beverages (n=9)	200 (170-300)	221 (133)	182 (112-282)	192 (124)	.16
	Oral nutritional supplements (n=3)	125 (50-225)	135 (96)	200 (220-225)	210 (22)	.11
	Soup (n=5)	135 (0-225)	117 (113)	111 (79-226)	144 (80)	.35
	Hot dishes (n=14)	390 (74-425)	296 (221)	329 (150-384)	296 (142)	.62
	Desserts (n=11)	80 (55-80)	75 (42)	55 (45-126)	80 (54)	.77
	Fruit (n=6)	13 (0-98)	43 (61)	63 (55-104)	80 (46)	.09
	Vegetables^c^ (n=10)	13 (0-71)	36 (47)	13 (0-71)	36 (18)	.88

^a^Differences between the methods are tested with Wilcoxon Signed Rank test.

^b^Includes butter/margarine/mayonnaise, sugary-based spreads, meat-based spreads, mayonnaise-based spreads, fish-based spreads, and cheese.

^c^Does not include vegetables as part of hot dishes.

**Table 5:** Proportions with 90% and 80% agreement between MyFood and the photograph method in estimated intake within food groups.

Food and beverage items		Percent agreement	
		90%	80%
**Day 1**			
	Bread and cereals (n=23)	22	57
	Spreads^a^ (n=22)	23	50
	Egg (n=11)	82	82
	Yogurt (n=9)	33	67
	Cold beverages (n=30)	57	77
	Hot beverages (n=9)	44	67
	Oral nutritional supplements (n=3)	67	67
	Soup (n=9)	22	56
	Hot dishes (n=20)	15	30
	Desserts (n=12)	8	42
	Fruit (n=10)	10	20
	Vegetables^b^ (n=10)	20	40
**Day 2**			
	Bread and cereals (n=24)	29	63
	Spreads^a^ (n=17)	53	71
	Egg (n=8)	88	88
	Yogurt (n=8)	50	75
	Cold beverages (n=25)	44	68
	Hot beverages (n=9)	44	67
	Oral nutritional supplements (n=3)	67	67
	Soup (n=5)	40	60
	Hot dishes (n=14)	14	36
	Desserts (n=11)	27	55
	Fruit (n=6)	0	17
	Vegetables^b^ (n=10)	10	20

^a^Includes butter/margarine/mayonnaise, sugary-based spreads, meat-based spreads, mayonnaise-based spreads, fish-based spreads, and cheese

^b^Does not include vegetables as part of hot dishes.

## Discussion

### Principal Findings

The MyFood app is developed for use among hospitalized patients at nutritional risk. According to the Norwegian Safety Program: “In Safe Hands” [31] all patients at nutritional risk should have a nutritional assessment, including dietary recording to compare intake against individual needs of energy, protein, and liquids. Further, nutrition-related measures should be performed and an individual nutrition plan created, before performing a reassessment after 3 days [31]. We found that 60%-80% of the participants had less than 20% disagreement between estimated intake of energy, protein, and liquids in MyFood and the photograph method. The agreement between the methods was higher the second recording day, compared to the first, and for the breakfast and lunch meal compared to the dinner. Recorded consumption of bread and cereals was higher in MyFood compared to the photograph method both days. Spreads; particularly butter, margarine, and mayonnaise, fruit, vegetables, and meal condiments were the food groups most often omitted by the patients. The majority of the patients rated MyFood as easy to use.

To our knowledge, no similar study has been conducted in patients to allow direct comparison of results.

### The Accuracy of MyFood’s Estimations of Energy, Protein, and Liquid on Group Level

Even though the main objective of the study was to evaluate recorded intake in MyFood on the individual level, estimated intake on the group level was analyzed to investigate if overall disagreement was present. The median total energy intake was not different between the methods the first recording day, however, a lower median intake in MyFood compared to the photograph method was found the second day. Underestimation of energy intake is often seen in validation studies of self-reporting dietary assessment tools among healthy adults [32] and among hospitalized patients [33]. This is also found for technology-based records [34,35]. MyFood’s target population is patients at nutritional risk who often have reduced food intake compared to needs [16]. Intentional underreporting of food intake may not be as relevant for our target population compared to healthy populations.

Median recorded protein intake was lower in MyFood compared to estimations from the photograph method in total, for breakfast, and for lunch recording day 1, and for breakfast recording day 2. This deviates from results in other validation studies on electronic dietary assessment tools. Raatz and coworkers [36] and Fukuo and colleagues [37] did not find a different recording of protein intake among healthy subjects in a personal digital assistant and a Web app, compared to 24-hour dietary recall and paper-based food records, respectively. Sliced bread with different types of spreads typically constitutes Norwegian breakfast and lunch meals, also in hospitals. Half of the participants had up to 20% disagreement in consumption of spreads between MyFood and the photograph method, and the individual drop-plots demonstrated that MyFood estimated lower intake of spreads compared to the photograph method for several participants on day 1. Spreads are often a protein source in bread-based meals. The agreement between the methods in intake of spreads was better the second day.

Recorded liquid intake in MyFood showed generally good agreement with the photograph method. However, recorded intake to the breakfast meal the first day was significantly lower compared to the photograph method. Several of the participants consumed both cold and hot beverages for the breakfast meal. This may have increased the chance of not remember to record all types of beverages consumed.

### The Accuracy of MyFood’s Estimations of Energy, Protein, and Liquid on Individual Level

The main aim of the present study was to evaluate MyFood’s ability to estimate the patients’ dietary intake on an individual level. This contrasts most other validation studies which focus on mean intake on group level and cross-classification, but not on absolute intakes. We evaluated MyFood compared to the photograph method for two separate days. A comparison of one-day and three-day calorie counts to estimate dietary intake by Breslow and Sorkin [38] suggested that 1-day calorie counts may be a valid alternative to the more labor-intensive 3-day count commonly performed in hospitalized patients. Førli and coworkers argue, however, that one day may be too short to estimate dietary intake among hospitalized patients [33]. The MyFood app is intended for use over several days, to follow-up dietary intake. This is in line with the common recommendation at Oslo University Hospital of using paper-based dietary assessment forms on a daily basis for patients at nutritional risk. The dietary recording in MyFood is more detailed than the paper-based forms used today by including a higher differentiation between type of meals and meal items. MyFood also includes more alternatives for portion sizes and provides the possibility to only record components of composite dishes. By these means there are reasons to assume that MyFood will provide a higher accuracy of the patient’s diet than the paper-based forms, if used correctly.

The individual drop-plots presented in the present study showed an overestimation of energy intake in MyFood, compared to the photograph method for some participants and underestimation for others. An explanation may be that both duplicate recordings and omissions of food items were observed. Five participants had duplicate recordings of some meal components the first day and one participant the second day. The largest discrepancies in energy intake between the methods on the individual level were found for the dinner meal the first day. This may be explained by inaccurate estimation of portion sizes for hot dishes. We found that several participants selected a full portion, even though not consuming a whole plate. The discrepancies in individual energy intake between the methods were wider the first recording day, and more coinciding the second day. We also observed fewer duplicate recordings in the app the second, compared to the first recording day. This may be due to a learning effect where the patients became more familiar with the app after one day of recording. A tendency to such a learning effect was observed in general in the evaluation study. A potential learning effect with the repeated use of a computerized dietary assessment tool was also found in a validation study among 41 adults with type 2 diabetes mellitus. The authors argued that the patients became more familiar with the website with repeated use [39].

We found a tendency to lower recorded protein intake in MyFood compared to the photograph method for participants with higher protein intake. This may be explained by the omission of typical protein-rich spreads with higher intakes due to recall bias. Cheese was omitted by three participants, ham by two participants, and egg by one participant. The second recording day the recorded protein intake in MyFood was more coinciding with the estimated protein intake in the photograph method.

Liquid consumption on individual level showed a tendency to increased deviations between MyFood and the photograph method among participants with a higher liquid intake. The first day the patient with the largest deviation had omitted both coffee and milk from the app recordings. The second day the intake recorded in MyFood was higher compared to the photograph method for some of the participants due to the recording of a full glass in the app, even though only consuming half or three-quarters of a full glass size.

The proportion of participants having less than 20% disagreement in MyFood and the photograph method was 69% for energy intake, 66% for protein intake, and 63% for liquid intake the first day, whereas the corresponding proportions were 76% for energy, 83% for protein and 72% for liquid intake the second day. This may be due to a learning effect as discussed above.

### The Accuracy of MyFood’s Estimation Within Food Groups

The majority of the food groups showed good agreement between MyFood and the photograph method on the group level. Good agreement in recording of food groups is consistent with findings from a validation study on a dietary assessment app for smartphones compared to repeated 24 h recall interviews [40] and Foodbook24; a Web-based dietary assessment tool [41]. The median intake of bread and cereals was higher in MyFood compared to the photograph method both recording days. Based on photograph observations and partial weighing this was found to be due to too large portion sizes of sliced bread and bread rolls in the app compared to the actual sizes served at the hospital. Recorded fruit intake was significantly lower in MyFood than consumption observed from photographs the first recording day. A possible explanation is that fruit intake was omitted by 27% of the participants on day 1. Medin and coworkers also found a high omission rate of fruits in a validation study among school children [30]. About 80% of the participants had more than 20% disagreement between estimated fruit consumption in MyFood and the photograph method both recording days. The majority of the participants’ fruit consumption was preprepared fruit boxes with sliced fruits. Based on the photograph method including observations and partial weighing we found the fruit boxes in the app to be disproportionately lower than the size most often observed and weighed. Revision of portion sizes for bread and cereals, and fruit cups, will probably lead to more accurate recordings of these food groups in the MyFood app.

In the present study, some of the standard portion sizes of hot dishes included in MyFood seemed to be too large compared to actual size served to the patients. A full portion size in MyFood was based on information from the hospital kitchen at OUH, Rikshospitalet on how standard portion sizes should be constituted when served. The visibility and description of what constitutes a full portion size in MyFood (Figure 2) may not have been clear enough for the patient. Several patients may have assumed eating a whole standard portion if the plates seemed full. In addition, studies have shown that small portion sizes tend to be overestimated and large portion sizes to be underestimated [42,43], and the former may have occurred in our study.

Twelve percent of foods and beverages were omitted in MyFood the first day, and 11% the second day. The food group most often omitted both recording days was butter, margarine, and mayonnaise. When recording several types of spreads, butter and margarine are typically easy to forget. Spreads were found to be among the food items most often omitted in a validation study of a Web-based dietary assessment tool among 117 school children [30]. The omission of food items in meals consisting of several secondary ingredients, like sandwiches, has been argued to be more common than in less composite meals [44]. Frequent omission of margarine was also found by Førli and colleagues [33] in a validation study of a self-administered dietary assessment form among 45 patients at OUH, Rikshospitalet. Prompting of questions related to the use of butter/margarine will be included in the further development of MyFood.

### Acceptance of Use by the Patients

The majority of the patients found MyFood easy to use and more than 70% became more aware of own nutritional needs. Electronic dietary assessment tools are generally well accepted and preferred over conventional methods among healthy subjects [46]. Our study population included patients ranging from 17 to 77 years with a mean age of 51 years. It is possible that use of an app for tablets is a larger barrier among older patients. However, qualitative studies among older persons have demonstrated that elderly persons often are positive to using tablets and eager to learn, even though cognitive deficits increase by age and low self-efficacy may limit the potential for use [47,48].

### Strengths and Limitations

The development process of the MyFood app involved nurses and patients, which is considered an important strength. The evaluation of recorded dietary intake in MyFood was compared to observations from meal photographs. The photograph method is a validated tool for assessment of dietary intake, compared to weighed records [45,49]. In addition, we combined the photograph observations with partial weighing of meal components which probably strengthened the method. Our photograph method is associated with different measurement errors than the dietary assessment functionality in MyFood, which is also considered an important strength. In addition to validate the MyFood app with regard to the accuracy of dietary recording we also investigated the users’ experiences with the tool. A recent scoping review on the use of technology in identifying hospital malnutrition highlighted the importance of establishing usability rating to determine the app’s actual usefulness in practical settings [50].

A limitation of the study is that only dietary intake to breakfast, lunch, and dinner was evaluated. Energy consumption for the breakfast, lunch, and dinner meals together have been reported to account for 85% of patients’ total intake [33]. By not including the total intake we do not know how accurate the dietary assessment function in MyFood estimate intake to snack meals, evening meals, and beverage intake in-between meals. Another potential limitation is that the patients knew that the researchers were taking photographs of their meals before and after consumption. This may have influenced their recording in the app by acting as a reminder. The evaluation study was performed among patients at a hematology and a gastrointestinal surgery department. We do not know whether our findings are representative for other groups of patients. The included patients were all sick, some quite severe. The presence of disease and fatigue may have influenced the precision of the recordings. MyFood is intended for use among patients at nutritional risk. Nutritional risk was, however, not an inclusion criterion in the present study, as we wanted to evaluate the app for patients with both small and larger food intake. Only patients with a certain food intake orally were included and we, therefore, do not know how the dietary assessment function in MyFood measures the intake for patients with tube feeding or parenteral nutrition.

### MyFood’s Potential for Use as a Dietary Assessment Tool Among Hospitalized Patients at Nutritional Risk

Based on the results in the present evaluation study, we consider MyFood as having good potential for use as a dietary assessment tool among hospitalized patients at nutritional risk. MyFood may provide support to health care workers in their tasks related to the nutritional treatment of patients at nutritional risk. This support may contribute to prevent development of disease-related malnutrition among at-risk patients. Corrections of some of the portion sizes in the app and prompting related to use of butter/margarine and portion size of dinner may increase the accuracy of the app further. An evaluation study among other patient groups may be valuable to amplify the potential for use of MyFood in the hospital setting.

### Conclusion

We have developed an app for tablets for use among hospitalized patients at nutritional risk. The app includes dietary assessment functionality for evaluation of patients’ dietary intake compared to individual needs of energy, protein, and liquids. The recorded intake of energy, protein, and liquids in MyFood showed good agreement with the photograph method for the majority of the participants. The app’s ability to estimate intake within food groups was good, except for bread and cereals which were overestimated, and fruit which was underestimated. MyFood was well accepted among the study participant and has the potential to be a dietary assessment tool for use among patients in clinical practice.

## Appendix

Multimedia Appendix 1 Selected screenshots from the MyFood app.

Multimedia Appendix 2 Drop plots illustrating individual intake of protein recording day 1 (n=32) and recording day 2 (n=29). Y-axis represents protein intake (grams). X-axis represents participant number ranged with increasing protein intake according to the photograph method. Equal protein intake from app and photograph observations is presented with only black dots.

Multimedia Appendix 3 Drop plots illustrating individual intake of liquid recording day 1 (n=32) and recording day 2 (n=29). Y-axis represents liquid intake (grams). X-axis represents participant number ranged with increasing liquid intake according to the photograph method. Equal liquid intake from app and photograph observations is presented with only black dots.

Multimedia Appendix 4 Drop plots illustrating individual intake of bread and cereals, and spreads recording day 1 and recording day 2. The Y-axis represents grams of food item. X-axis represents participant number ranged with increasing intake according to the photograph method. Equal food intake from app and photograph observations is presented with only white dots.

Multimedia Appendix 5 Drop plots illustrating individual intake of hot dishes and cold beverages recording day 1 and recording day 2. The Y-axis represents grams of food or beverage item. X-axis represents participant number ranged with increasing intake according to photograph observations. Equal food intake from app and photograph observations is presented with only white dots.

Multimedia Appendix 6 Number of food and beverage items observed from photographs and recorded in MyFood the first recording day. One item means one type of food or beverage in each meal.

Multimedia Appendix 7 Number of food and beverage items observed from photographs and recorded in MyFood the second recording day. One item means one type of food or beverage in each meal.

## Abbreviations

BMI: body mass index
IOR: interobserver reliability
NPR: Norwegian patient registry
OUH: Oslo University Hospital
TSD: services for sensitive data
USIT: University Center for Information Technology
