# Supplementary figures and images for: A Dietary Assessment App for Hospitalized Patients at Nutritional Risk: Development and Evaluation of the MyFood App
Source: JMIR Mhealth Uhealth. 2018 Sep 7;6(9):e175. doi: 10.2196/mhealth.9953 (PMC6231855; doi:10.2196/mhealth.9953)

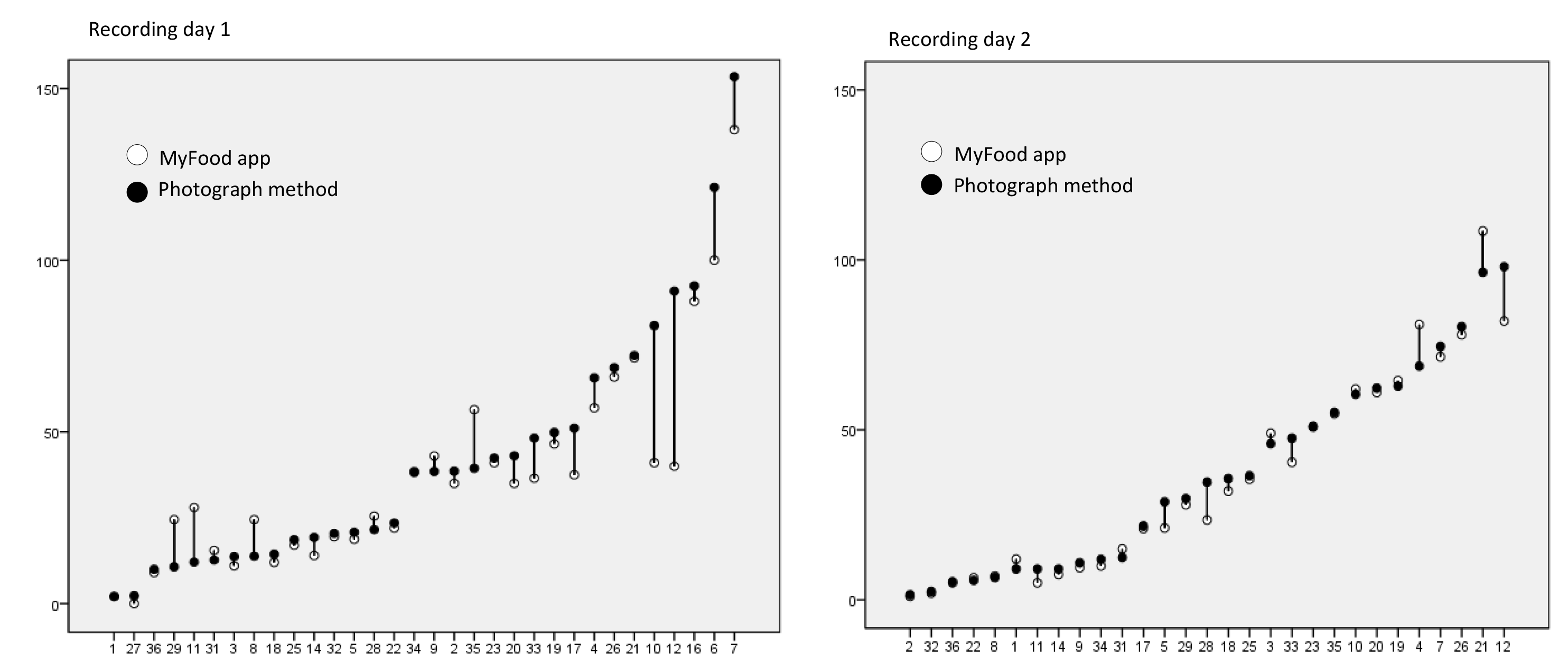

Supplement: Multimedia Appendix 2 [file mhealth_v6i9e175_app2.png]

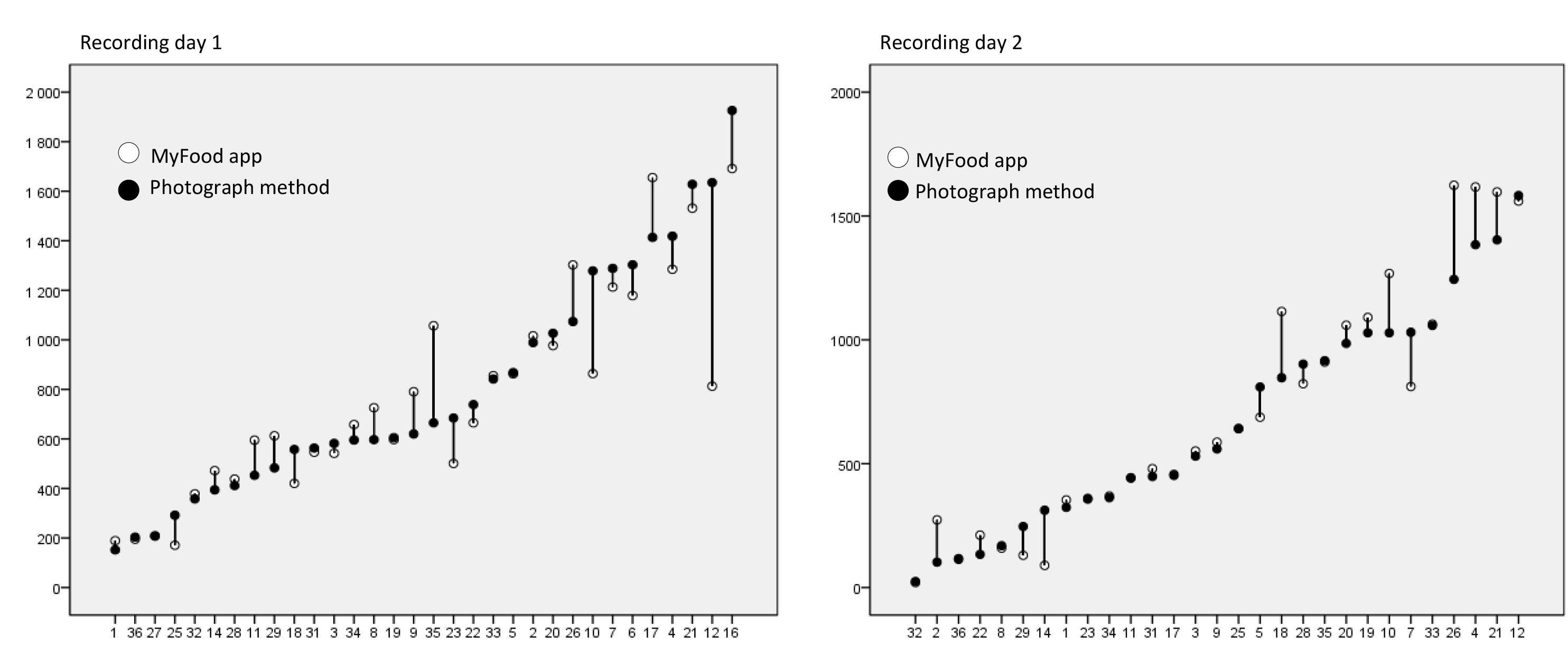

Supplement: Multimedia Appendix 3 [file mhealth_v6i9e175_app3.png]

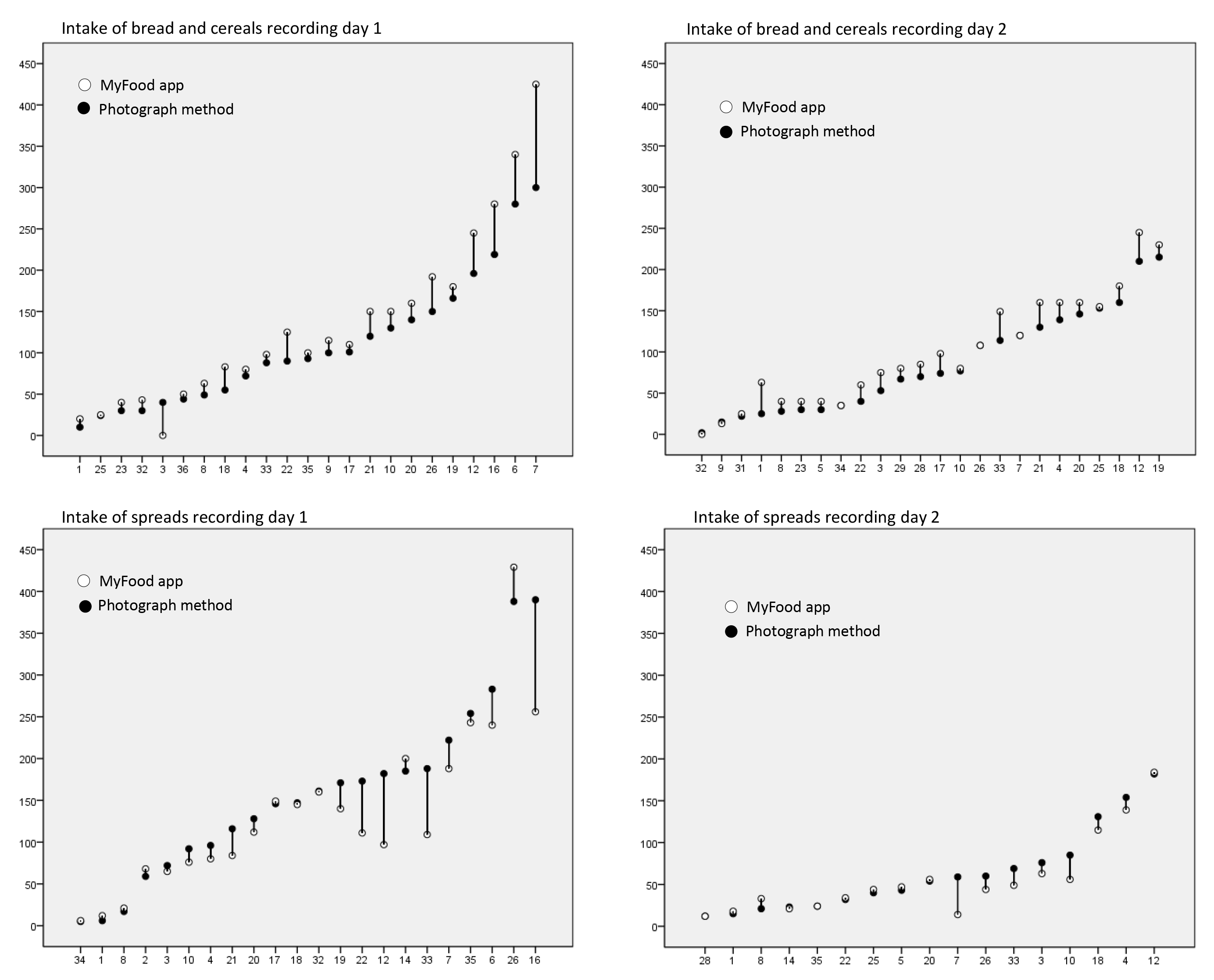

Supplement: Multimedia Appendix 4 [file mhealth_v6i9e175_app4.png]

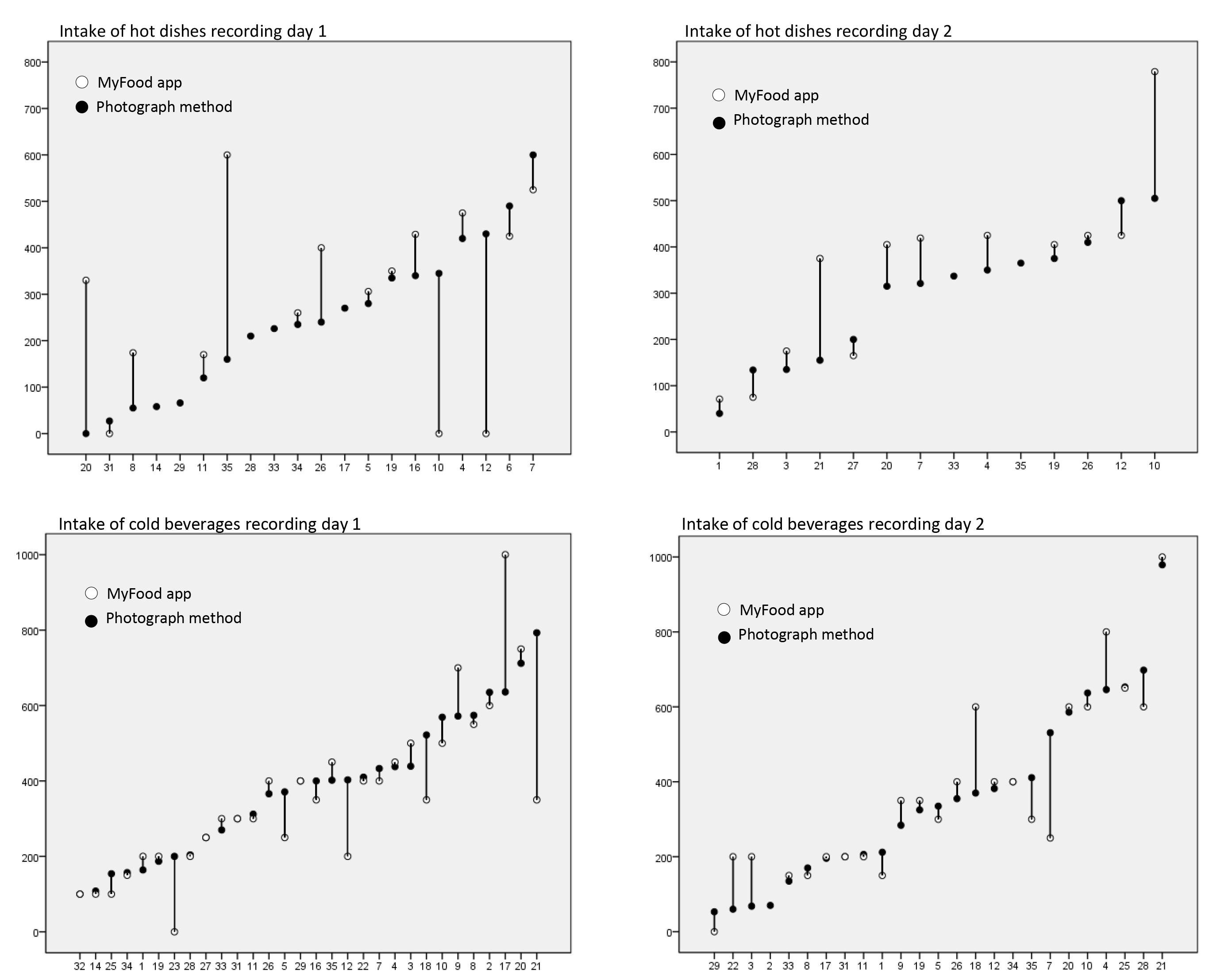

Supplement: Multimedia Appendix 5 [file mhealth_v6i9e175_app5.png]

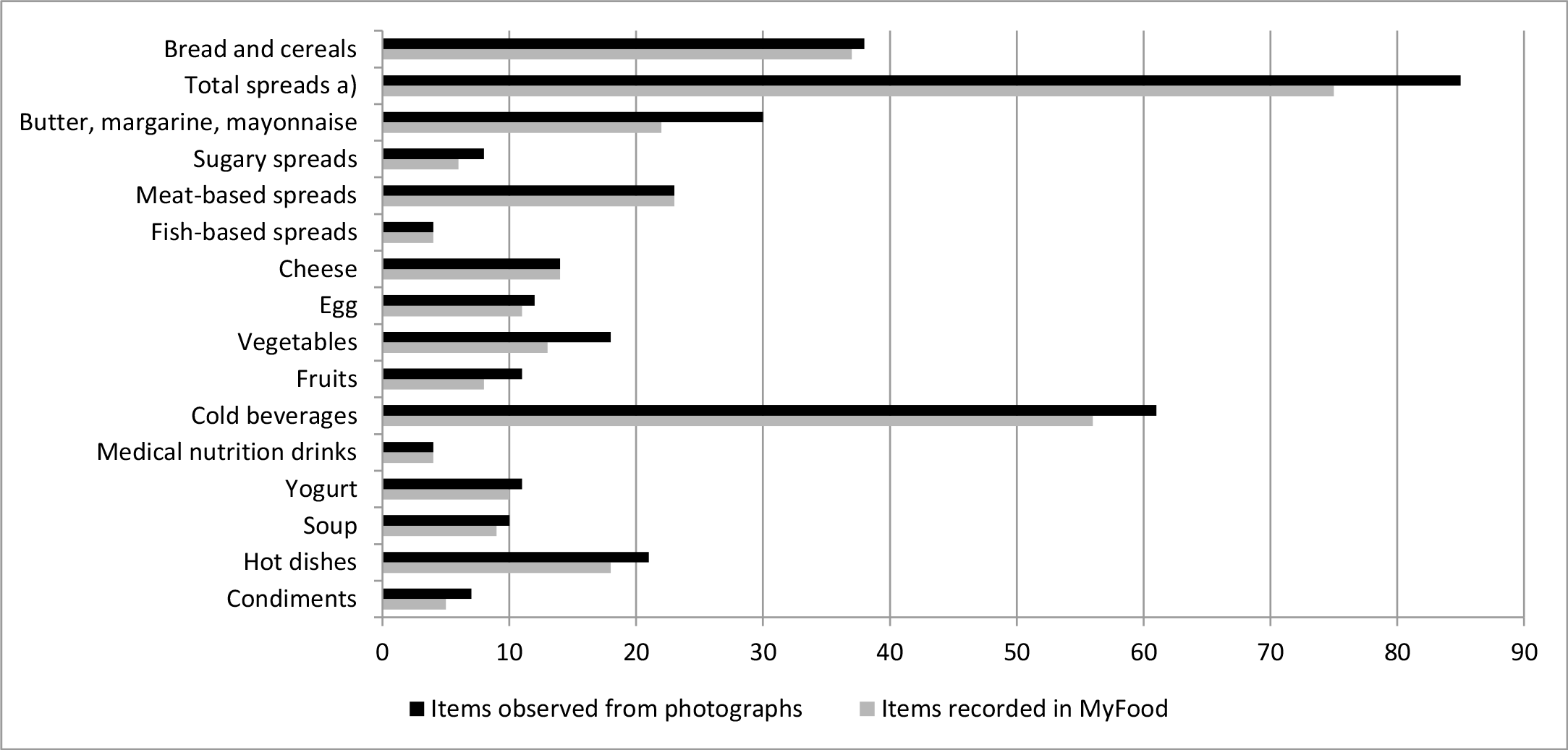

Supplement: Multimedia Appendix 6 [file mhealth_v6i9e175_app6.png]

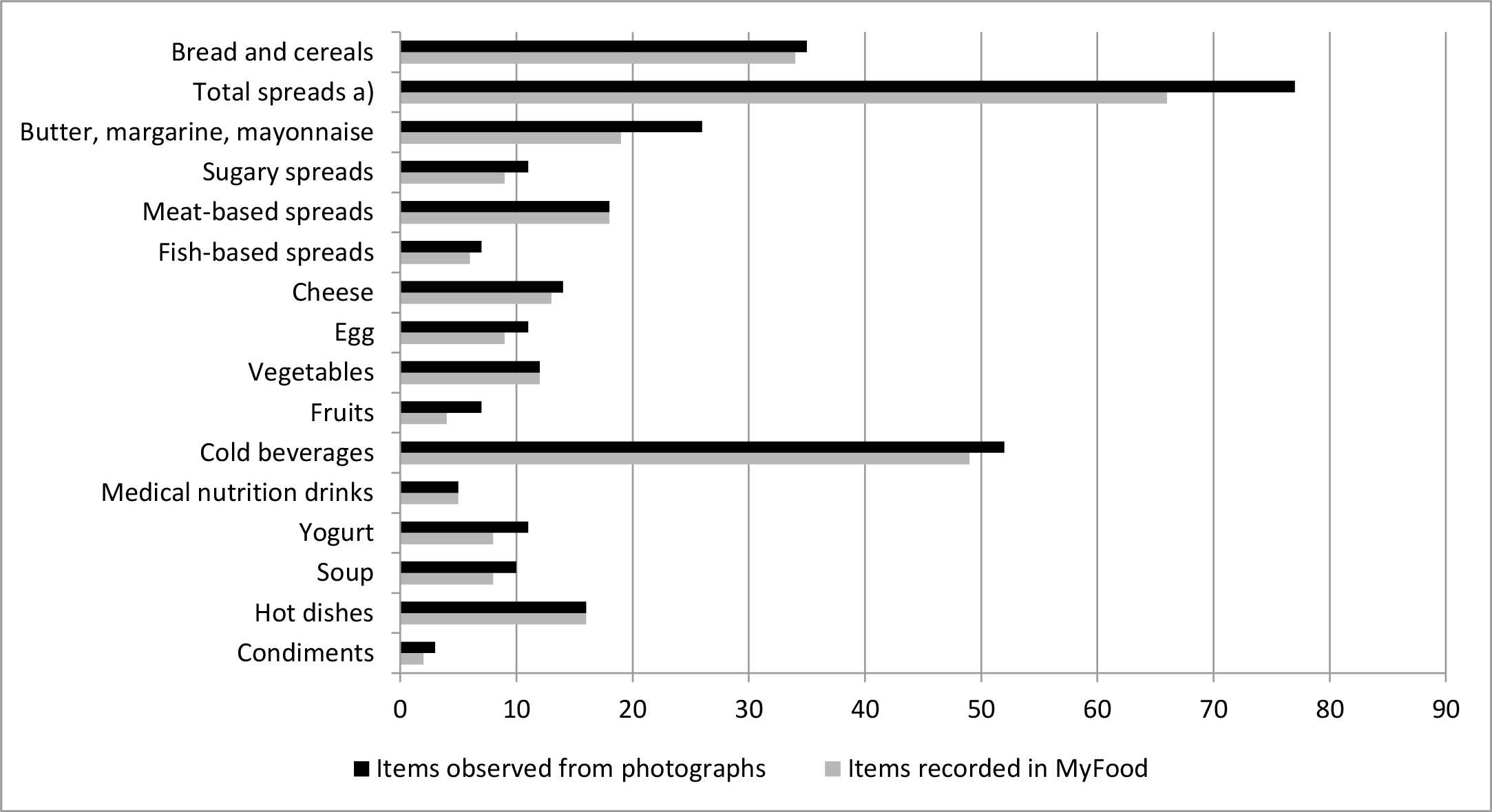

Supplement: Multimedia Appendix 7 [file mhealth_v6i9e175_app7.png]
